# 

**Published:** 1895-07

**Authors:** 


					﻿WHEN WRITING TO ADVERTISERS, PLEASE SAY “I SAW^YQUR
AD. IN THE ATLANTA MEDICAL AND SURGICAL JOURNAL.’^''
______________________________________________
CtTABtlSHEO 1855.	SUBSCRIPTION. $2.00.
ATLANTA
IWEDIGflIi flHD SURGIGflIt
JOURNAL.
LUTHER B. GRANDY, M.D.,	JI. B. HUTCHINS, JI.D.,
MANAGING EDITOR.	BVSINE.S.S MANAGER.
CfJLLABORATORS :
H. V. M. 3IILLEK, M.D., LL.D., VIRGIL O. HARDOX, M.D., FLOYD W. McRAE, M.D.,
DUNBAR ROY, M.D., and H. R. SLaUK, M.D., 1’h.M.
NEwsERiEi; Atla.nta, Ga., July, 1895.	No. 5.
NERVOUS
PROSTRATION
CHAS. ROOME PARMELE CO., 98 WILLIAM STu N.Y< 1
PUBI.ISHBD BT THE FRANKLIN PRINTING AND PUBLISHING CO., Atlanta, Ga.
Eoteted at the‘Pdst-Office at AtlUata, Gft., aEfsecond-claM matter.
				

